# Identification of Repellent and Insecticidal Constituents of the Essential Oil of *Artemisia rupestris* L. Aerial Parts against *Liposcelis bostrychophila* Badonnel

**DOI:** 10.3390/molecules180910733

**Published:** 2013-09-03

**Authors:** Xin Chao Liu, Yin Ping Li, He Qin Li, Zhi Wei Deng, Ligang Zhou, Zhi Long Liu, Shu Shan Du

**Affiliations:** 1 Department of Entomology, China Agricultural University, Haidian District, Beijing 100193, China; E-Mails: xchliu@yeah.net (X.C.L.); hqliaau@163.com (H.Q.L.); 2 Analytical and Testing Center, Beijing Normal University, Haidian District, Beijing 100875, China; E-Mails: xinjiang20041021@163.com (Y.P.L.); dengzw@bnu.edu.cn (Z.W.D.); 3 Department of Plant Pathology, China Agricultural University, Haidian District, Beijing 100193, China; E-Mail: lqzhou@cau.edu.cn; 4 State Key Laboratory of Earth Surface Processes and Resource Ecology, Beijing Normal University, Beijing 100875, China

**Keywords:** *Liposcelis bostrychophila*, *Artemisia rupestris*, contact toxicity, fumigant toxicity, repellency, essential oil composition

## Abstract

The aim of this research was to determine the chemical composition and insecticidal and repellent activity of the essential oil of *Artemisia rupestris* L. aerial parts against the booklice *Liposcelis bostrychophila* Badonnel and isolation of insecticidal and repellent constituents from the essential oil. The essential oil of *A. rupestris* was obtained by hydrodistillation and analyzed by GC-MS. A total of 30 components of the essential oil of *A. rupestris* was identified and the principal compounds in the essential oil were α-terpinyl acetate (37.18%), spathulenol (10.65%), α-terpineol (10.09%), and linalool (7.56%), followed by 4-terpineol (3.92%) and patchoulol (3.05%). Based on bioactivity-guided fractionation, the four active constituents were isolated from the essential oil and identified as α-terpineol, α-terpinyl acetate, 4-terpineol and linalool. The essential oil of *A. rupestris* exhibited contact toxicity against *L. bostrychophila* with LD_50_ value of 414.48 µg/cm^2^. α-Terpinyl acetate (LD_50_ = 92.59 µg/cm^2^) exhibited stronger contact toxicity against booklice than α-terpineol (LD_50_ = 140.30 µg/cm^2^), 4-terpineol (LD_50_ = 211.35 µg/cm^2^), and linalool (LD_50_ = 393.16 µg/cm^2^). The essential oil of *A. rupestris* (LC_50_ = 6.67 mg/L air) also possessed fumigant toxicity against *L. bostrychophila* while the four constituents, 4-terpineol, α-terpineol, α-terpinyl acetate and linalool had LC_50_ values of 0.34, 1.12, 1.26 and 1.96 mg/L air, respectively. α-Terpinol and α-terpinyl acetate showed strong repellency against *L. bostrychophila*, while linalool and 4-terpinol exhibited weak repellency. The results indicate that the essential oil of *A. rupestris* aerial parts and its constituent compounds have potential for development into natural insecticides or fumigants as well as repellents for control of insects in stored grains.

## 1. Introduction

Booklice (*Liposcelis bostrychophila* Badonnel; Psocoptera: Liposcelididae) have a worldwide distribution infesting domestic premises, raw material stores, manufacturing factories, and historical documents in museums [1]. Booklice do not bite, or transmit disease and are considered as nuisance pests rather than a cause of losses of stored commodities [2]. Booklice were generally regarded as secondary pests, often overlooked due to their small size and the existence of other more damaging post-harvest primary pests [e.g., maize weevils (*Sitophilus zeamais* Motschulsky), rice weevils (*S. oryzae* L.) and lesser grain borer (*Rhyzopertha dominica* Fabricius)] in cereal grains. However, new evidence indicates that psocids are perhaps the most important emerging pests in stored grains and related commodities due to their small size, and resistance to chemicals [1]. Currently, recommended pest control measures in durable stored food products rely heavily on use of synthetic insecticides/fumigants which pose possible health hazards to warm-blooded animals, risk of environmental pollution, development of resistance by insects and pest resurgence [3] These problems have necessitated a search for alternative eco-friendly insect pest control methods. In recent years, global research has focused on the possible use of essential oils, in protection of stored agricultural products [4,5]. Investigations in several countries confirm that some plant essential oils not only repel insects, but possess contact and fumigant toxicity against stored product pests as well as exhibiting feeding inhibition or harmful effects on the reproductive system of insects [4,5,6].

During the screening program for new agrochemicals from Chinese medicinal herbs and wild plants, the essential oil of *Artemisia rupestris* L. aerial parts was found to possess insecticidal toxicity against booklice (*L. bostrychophila*). The genus *Artemisia* belongs to the family Compositae (Asteraceae) and is a large, diverse genus of plants with about 380 species around the World, of which 186 species (82 endemic) are distributed in China [7]. Many *Artemisia* species are rich in polyacetylenes, flavonoids, terpenoids, and cyanogenic glycosides and are well-known medicinal plants. Rock wormwood (*A. rupestris*) is a perennial subshrub distributed in dry hills, desert or semi-desert steppes as well as dry river valleys (1,100–2,900 m) in the Xinjiang Uyghur Autonomous Region of China and also distributed in Afghanistan, Kazakhstan, Kyrgyzstan, Mongolia, Russia, Tajikistan; East and North of Europe [7]. *A. rupestris* is a well-known traditional Chinese medicinal plant in the Xinjiang of China used for detoxification, with antitumor, antibacterial, and antivirus properties, and is used as well for protecting the liver [8]. Several alkaloids, sesquiterpenoids, sesquiterpene lactone glycosides, triterpenoids, and flavonoids have been identified from *A. rupestris* [9,10,11,12,13,14,15,16,17]. The chemical composition of the essential oil derived from *A. rupestris* aerial parts has been determined previously [18,19,20]. Essential oils of several Chinese *Artemisia* species were demonstrated to possess insecticidal activity to grain storage insects [21,22,23,24,25,26,27,28]. However, a literature survey shows that there is no report on insecticidal activity of the essential oil derived from *A. rupestris* aerial parts. Thus, the objective of this study was to investigate the chemical constituents and insecticidal activity of the essential oil of *A. rupestris* aerial parts against the booklice and isolation of active constituent compounds from the essential oil. 

## 2. Results and Discussion

### 2.1. Essential Oil Chemical Composition

The yellow essential oil yield of *A. rupestris* aerial parts was 0.21% (V/W) and the density of the concentrated essential oil was determined as 0.88 g/mL. A total of 30 components of the essential oil of *A. rupestris* were identified, accounting for 98.01% of the total oil (Table 1). The principal compounds in the essential oil were α-terpinyl acetate (37.18%), spathulenol (10.65%), α-terpineol (10.09%), and linalool (7.56%) followed by 4-terpineol (3.92%) and patchoulol (3.05%). Monoterpenoids represented 19 of the 30 compounds, corresponding to 72.97% of the whole oil, while nine of the 30 constituents (23.86% of the crude essential oil) were sesquiterpenoids. The chemical composition of the essential oil of *A. rupestris* aerial parts in the present study was different from that reported in previous studies. For example, α-terpinyl acetate (18.68%), β-myrcene (15.95%), terpinolene (8.59%), β-terpineol (5.77%) and alloocimene (4.43%) were the previously reported major compounds in the essential oil of *A. rupestris* [18], however, the two main constituents of the essential oil derived from *A. rupestris* were dihydrocarvyl acetate (31.54%) and 2-hydroxy-3-(1-propenyl group)-1,4-napthoquinone (27.28%) [20]. The essential oil from European *A. rupestris* contained hexadecanol (18.1%), hexadecanoic acid (11.2%), guaiol (6.2%) and β-elemene (3.9%) [19]. In the previous reports, influence of environmental factors on *p*-cymene, γ-terpinene, linalool and thymol levels in the essential oil of *Thymus migricus* was evident [29] and monoterpenes and sesquiterpene hydrocarbons in the essential oils of *Lychnophora ericoides* were strongly related to chemical balance in soils (organic matter, P and base saturation) [30]. The above findings demonstrated that there are broad differences in the chemical components for *A. rupestris*. Thus, further studies on plant cultivation and essential oil standardization are necessary because chemical composition of the essential oil of *A. rupestris* varies greatly with the plant population as well as harvest time. 

**Table 1:** Chemical constituents of the essential oil derived from *Artemisia rupestris* aerial parts.

Peak No.	RI *	Compound	Composition, %
1	967	Sabinene	0.06
2	981	β-Pinene	0.19
3	992	Myrcene	1.47
4	1018	(+)-4-Carene	0.19
5	1023	β-Cymene	0.11
6	1030	D-Limonene	0.28
7	1032	1,8-Cineol	0.39
8	1067	*cis*-Linalool oxide	0.19
9	1094	Linalool	7.56
10	1175	4-Terpineol	3.92
11	1188	α-Terpineol	10.09
12	1204	Verbenone	0.61
13	1227	Citronellol	2.23
14	1255	Geraniol	2.01
15	1285	Bornyl acetate	0.62
16	1340	Linalyl propionate	2.62
17	1349	α-Cubebene	0.69
18	1351	α-Terpinyl acetate	37.18
19	1355	Citronellol acetate	2.14
20	1356	Eugenol	1.18
21	1391	β-Elemene	0.85
22	1420	β-Caryophyllene	1.80
23	1453	Geranyl acetone	1.11
24	1457	( *Z*)-β-Farnesene	0.61
25	1494	α-Selinene	1.07
26	1523	δ-Cadinene	1.36
27	1578	Spathulenol	10.65
28	1583	Caryophyllene oxide	2.09
29	1663	Patchoulol	3.05
30	1672	Valeranone	1.59
		Total identified	98.01
		Monoterpenoids	72.97
		Sesquiterpenoids	23.86
		Others	1.18

* RI, retention index as determined on a HP-5MS column using the homologous series of *n*-hydrocarbons.

### 2.2. Insecticidial Activities

The essential oil of *A. rupestris* aerial parts exhibited contact toxicity against *L. bostrychophila* with LD_50_ value of 418.48 µg/cm^2^ (Table 2). When compared with the positive control pyrethrum extract (LD_50_ = 18.99 µg/cm^2^), the essential oil demonstrated 22 times less toxicity to *L. bostrychophila*. Four constituent compounds, α-terpinyl acetate, α-terpineol, 4-terpineol acetate and linalool exhibited contact toxicity against the booklice, with LD_50_ values of 92.59, 140.30, 211.35 and 393.16 µg/cm^2^, respectively (Table 2). α-Terpinyl acetate had almost 4.5 times more toxicity than the crude essential oil against the booklice. Moreover, α-terpinyl acetate and α-terpineol possessed almost four and three times more toxicity than linalool against booklice, respectively. It is suggested that α-terpinyl acetate and α-terpineol are major contributors to the insecticidal (contact) activity of the essential oil. However, compared with pyrethrum extract (positive control), α-terpinyl acetate showed only five times less toxicity against booklice.

**Table 2:** Contact toxicity and fumigant toxicity of the essential oil of *Artemisia rupestris* aerial parts and its constituents against *Liposcelis bostrychophila*.

Toxicity	Treatment	LD_50_ LC_50_	95% FL *	Slope ± SE	Chi square (χ^2^ )
Contact Toxicity (μg/cm^2^)	*A. rupestris*	418.48	389.34–447.09	8.57 ± 0.83	17.92
	Linalool	393.16	368.45–429.53	9.86 ± 0.91	13.76
	α-Terpineol	140.30	129.67–151.29	5.08 ± 0.48	18.48
	α-Terpinyl acetate	92.59	85.12–100.45	7.29 ± 0.74	12.88
	4-Terpineol	211.35	196.27–230.65	8.74 ± 0.83	15.47
	Pyrethrum extract	18.99	17.56–20.06	7.64 ± 1.05	-
Fumigant (mg/L air)	*A. rupestris*	6.67	6.21–6.98	7.03 ± 0.63	16.24
	Linalool	1.96	1.85–2.12	5.78 ± 0.56	12.33
	α-Terpineol	1.12	1.01–1.21	4.11 ± 0.34	14.56
	α-Terpinyl acetate	1.26	1.14–1.33	5.49 ± 0.51	10.16
	4-Terpineol	0.34	0.31–0.38	4.13 ± 0.33	12.19
	Dichlorvos	1.35 × 10^−3^	1.25–1.47 × 10^−3^	6.87 ± 0.77	-

* Fiducial limits.

4-Terpineol (LC_50_ = 0.34 mg/L air) exhibited stronger fumigant toxicity against *L. bostrychophila* adults than α-terpineol (LC_50_ = 1.12 mg/L air), α-terpinyl acetate (LC_50_ = 1.26 mg/L air) and linalool (LC_50_ = 1.96 mg/L air), while the crude essential oil of *A. rupestris* aerial parts showed an LC_50_ value of 6.67 mg/L air (Table 2). 4-Terpineol showed almost 20 times stronger fumigant toxicity than the essential oil against the booklice. However, compared with the positive control, dichlorvos (LC_50_ = 1.35 × 10^−3^ mg/L air), α-terpineol and the crude essential oil exhibited almost 250 times and 4,940 times less toxicity to *L. bostrychophila*, respectively. Moreover, in the previous studies, the two active constituent compounds, 4-terpineol and α-terpineol have been found to possess strong fumigant toxicity against several grain storage insects, such as *S. granaries* L., *S. oryzae*, *S. zeamais*, *Tribolium* *castaneum* Herbst, *T. confusum* L., and *R. dominica* [31,32,33,34,35,36,37,38]. α-Terpinyl acetate was regarded as active termiticidal compounds of hinoki wood (*Chamaecyparis obtusa*) [39]. However, there is no report on fumigant and contact toxicity of α-terpinyl acetate against grain storage insects after literature survey. Another constituent compound, linalool was also shown to exhibit fumigant toxicity against several insects. e.g., *S. zeamais* [40], the triatomine bug (*Rhodnius prolixus*) [41] and houseflies with a 24 h LC_50_ value of 13.6 mg/L air [42]. Moreover, linalool possessed both contact and fumigant toxicity against human head louse (*Pediculus humanus capitis*) [43] and exhibited a high acaricidal activity by vapor action against mobile stages of *Tyrophagus putrescentiae* [44]. 4-Terpineol, α-terpineol and linalool were found to be a competitive inhibitor of acetyl-cholinesterase (AchE) [45]. The above findings suggest that insecticidal activity especially fumigant activity of the essential oil of *A. rupestris* aerial parts and its four constituent compounds against the booklice is quite promising. As currently used fumigants are synthetic insecticides and the most effective fumigants (e.g., phosphine and methyl bromide) are also highly toxic to humans and other non-target organisms, the essential oil of *A. rupestris* aerial parts and its four constituent compounds show potential to be developed as possible natural fumigants/insecticides for the control of *L. bostrychophila*.

In traditional Chinese medicine, *A. rupestris* aerial parts are used for detoxification and for protecting the liver [8]. Moreover, many pharmacological activities of *A. rupestris* were demonstrated such as anti-tumor, antioxidant, antibacterial, and antivirus properties [8]. It thus seems that this medicinal herb is quite safe for human consumption because it has been used as a medicinal herb for hundreds of years. However, no experimental data about the safety of this herb is available so far, so to develop a practical application for the essential oil and the isolated constituents as novel fumigants/insecticides, further research on the safety of the essential oil/compounds to humans is needed. Additional studies on the development of formulations are also necessary to improve the efficacy and stability and to reduce cost.

### 2.3. Repellency

The results of repellency assays for the essential oil of *A. rupestris* aerial parts and isolated constituents against *L. bostrychophila* are presented in Table 3. Data showed that at tested concentrations, α-terpinol and terpinyl acetate showed strong repellency against *L. bostrychophila*. At the lowest assayed concentration (3.2 nL/cm^2^), the two compounds still showed moderate (Class III) repellency (52% and 48%, respectively) against the booklice at 4 h after exposure (Table 3). Linalool and 4-terpinol also exhibited weak repellency against *L. bostrychophila*. At the highest concentration (13 nL/cm^2^), the compounds exhibited only 53% and 46% (Class III) repellency against *L. bostrychophila* at 4 h after exposure, respectively (Table 3). Compared with the positive control,

**Table 3:** Percentage repellency (PR) after two exposure times for the essential oil and isolated constituents against *Liposcelis bostrychophila* *.

Treatment	2 h			4 h		
	13 nL/cm^2^	6.4 nL/cm^2^	3.2 nL/cm^2^	13 nL/cm^2^	6.4 nL/cm^2^	3.2 nL/cm^2^
DEET	100 ± 0a	84 ± 7a	72 ± 9a	94 ± 2a	82 ± 6a	64 ± 5a
Oil	76 ± 8c	56 ± 4b	41 ± 6b	73 ± 6c	42 ± 9c	29 ± 7cd
Linalool	72 ± 5cd	42 ± 9c	27 ± 6c	53 ± 9d	31 ± 6d	23 ± 9d
α-Terpinol	85 ± 4bc	75 ± 4a	69 ± 9a	84 ± 6b	65 ± 7b	52 ± 7ba
4-Terpinol	64 ± 8d	55 ± 5b	40 ± 5b	46 ± 7d	47 ± 7c	32 ± 9c
Terpinyl acetate	91 ± 7ab	76 ± 7a	62 ± 7a	86 ± 4b	67 ± 9b	48 ± 9b

* means in the same column followed by the same letters do not differ significantly (*P* > 0.05) in ANOVA and Tukey’s tests. PR was subjected to an arcsine square-root transformation before ANOVA and Tukey’s tests.

DEET and terpinyl acetate exhibited the same level of repellency against *L. bostrychophila* at 2 h after exposure and at 4 h after exposure, DEET and α-terpinol had the same level of repellency (Table 3). However, the two other constituents, linalool and 4-terpinol showed less repellency against the booklice. Many essential oils and their constituents had been evaluated for repellency against insects [46]. However, only few reports on the booklouse [47,48,49]. In this paper, we report to isolate four insecticidal and repellent constituents from the essential oil of *A. rupestris* aerial part against the booklice for the first time. These findings suggest that the essential oil of *A. rupestris* aerial parts and the four isolated compounds show potential for development as natural repellents or insecticides for stored product insects.

## 3. Experimental

### 3.1. General

^1^H and ^13^C-NMR spectra were recorded on Bruker ACF300 [300 MHz (1H)] and AMX500 [500 MHz (1H)] instruments using CDCl_3_ as the solvent with TMS as internal standard. EIMS were determined on a ThermoQuest Trace 2000 mass spectrometer at 70 eV (probe). Components of the essential oil of *A. rupestris* aerial parts were separated and identified by gas chromatography-flame ionization detection (GC-FID) and gas chromatography–mass spectrometry (GC–MS) on an Agilent 6890N gas chromatograph equipped with a flame ionization detector connected to an Agilent 5973N mass selective detector. The same capillary column (HP-5MS 30 m × 0.25 mm × 0.25 μm) and analysis conditions were used for both GC-FID and GC-MS.

### 3.2. Plant Material and Essential Oil Extraction

The dried aerial parts of *A. rupestris* (5 kg) were purchased from Anguo Chinese Medicinal Herbs Market (Anguo 071200, Hebei, China). The plant was identified by Dr. Liu, QR (College of Life Sciences, Beijing Normal University, Beijing, China) and a voucher specimen (CMH-Xinjiangyizhihao-Xinjiang-2013-04) was deposited in the museum of Department of Entomology, China Agricultural University. The sample was ground to a powder using a grinding mill (Retsch Muhle, Haan, Germany). The powder was subjected to hydrodistillation using a modified Clevenger-type apparatus for 6 h and extracted with *n*-hexane. Anhydrous sodium sulphate was used to remove water after extraction. The essential oil was stored in airtight containers in a refrigerator at 4 °C for subsequent experiments.

### 3.3. Insects

Booklice, *L. bostrychophila* were obtained from laboratory cultures in the dark in incubators at 28–30 °C and 70%–80% relative humidity and was reared on a 1:1:1 mixture, by mass, of milk powder, active yeast, and flour. All the containers housing insects and the Petri dishes used in experiments were made escape proof with a coating of polytetrafluoroethylene (Fluon^®^, Blades Biological, Edenbridge, UK). Laboratory bioassays were done within one week after adult collections.

### 3.4. Gas Chromatography-Mass Spectrometry

The GC settings were as follows: the initial oven temperature was held at 60 °C for 1 min and ramped at 10 °C min^−1^ to 180 °C where it was held for 1 min, and then ramped at 20 °C min^−1^ to 280 °C and held there for 15 min. The injector temperature was maintained at 270°C. The samples (1 μL, diluted to 1:100 in acetone) were injected, with a split ratio of 1:10. The carrier gas was helium at flow rate of 1.0 mL·min^−1^. Spectra were scanned from 20 to 550 *m/z* at 2 scans s^−1^. Most constituents were identified by gas chromatography by comparison of their retention indices with those of the literature or with those of authentic compounds available in our laboratories. The retention indices were determined in relation to a homologous series of *n*-alkanes (C_8_–C_24_) under the same operating conditions. Further identification was made by comparison of their mass spectra with those stored in NIST 05 (Standard Reference Data, Gaithersburg, MD, USA) and Wiley 275 libraries (Wiley, New York, NY, USA) or with mass spectra from the literature [50]. Component relative percentages were calculated based on GC peak areas without using correction factors.

### 3.5. Bioassay-Directed Fractionation

The crude essential oil of *A. rupestris* aerial parts (20 mL) was chromatographed on a silica gel (Merck 9385, 1,000 g) column (85 mm i.d., 850 mm length) by gradient elution with a mixture of solvents (*n*-hexane, *n*-hexane-ethyl acetate). Fractions (500 mL) were collected and concentrated at 40 °C, and similar fractions according to thin layer chromatography (TLC) profiles were combined to yield 15 fractions. Fractions (3–5, 7–9) that possessed contact toxicity, with similar TLC profiles, were pooled and further purified by preparative silica gel column chromatography (PTLC) until obtain the pure compound for determining structure as linalool (**1**, 0.6 g), 4-terpineol (**2**, 0.3 g), α-terpineol (**3**, 0.8 g), and α-terpinyl acetate (**4**, 1.2 g). The structure of the compounds was elucidated based on high-resolution electron impact mass spectrometry and nuclear magnetic resonance. 

### 3.6. Isolated Constituent Compounds

**Figure 1:**
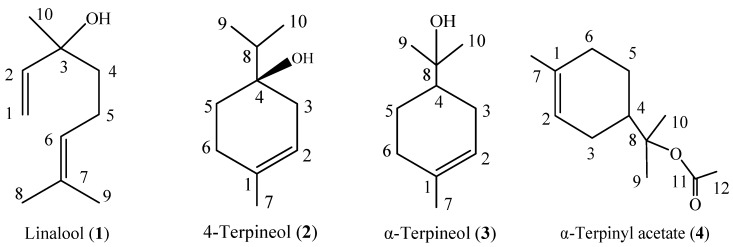
Constituent compounds isolated from the essential oil of *Artemisia rupestris* aerial parts.

*Linalool* (**1**, Figure 1). Colorless oil, ^1^H-NMR (CDCl_3_, 500 MHz) δ (ppm): 5.92 (1H, dd, *J* = 10.0, 10.0 Hz, H-2), 5.22 (1H, d, *J* = 20.0 Hz, H-1, *trans*), 5.12 (1H, t, H-6), 5.06 (1H, d, *J* = 10.0 Hz, H-1, *cis*), 2.03 (2H, m, 5-CH_2_), 1.83 (1H, br, 3-OH, which was removed in the deuterium exchange spectrum after adding a drop of deuterated water), 1.68 (3H, s, 9-CH_3_), 1.60 (3H, s, 8-CH_3_), 1.53 (2H, m, 4-CH_2_), 1.27 (3H, s, 10-CH_3_). ^13^C-NMR (CDCl_3_, 125 MHz) δ (ppm): 145.05 (C-2), 131.87 (C-7), 124.44 (C-6), 111.65 (C-1), 73.43 (C-3), 41.57 (C-4), 27.83 (C-10), 25.67 (C-8), 22.71 (C-5), 17.66 (C-9). EI-MS *m/z* (%): 154 (M^+^, 5), 136 (15), 121 (25), 109 (11), 93 (80), 83 (18), 80 (30), 71 (100), 69 (50), 55 (45), 43 (39), 41(62), 27 (15). The data matched with previous reports [40,51].

*4-Terpineol* (**2**, Figure 1). Colorless oil, ^1^H-NMR (CDCl_3_, 500 MHz) δ (ppm): 5.31 (1H, dd, *J* = 5.0 Hz, 2.5 Hz, H-2), 2.18 (2H, dd, *J* = 15.0 Hz, 5.0 Hz, H-3), 1.94 (2H, dd, *J* = 15.0 Hz, 5.0 Hz, H-6), 1.72 (3H, m, H-7), 1.66 (1H, m, H-8), 1.58 (2H, m, H-5), 0.96 (3H, d, *J* = 10.0 Hz, H-9), 0.94 (3H, d, *J* = 5.0 Hz, H-10). ^13^C-NMR (CDCl_3_, 125 MHz) δ (ppm): 133.86 (C-1), 118.45 (C-2), 71.74 (C-4), 36.78 (C-8), 34.61 (C-5), 30.80 (C-3), 27.06 (C-6), 23.28 (C-7), 6.85 (C-9); 6.82 (C-10). EI-MS *m/z* (%): 154 (M^+^, 15), 111 (50), 93 (44), 86 (23), 71 (100), 69 (21), 68 (15), 55 (17), 43 (29), 41(23). The data matched with previous reports [40,51].

α-*Terpineol* (**3**, Figure 1). Colorless oil, ^1^H-NMR (CDCl_3_, 500 MHz) δ (ppm): 5.38–5.44 (1H, m, H-2), 2.11 (1H, d, *J* = 8.4 Hz, H-3), 2.01 (1H, d, *J* = 5.0 Hz, H-6), 1.89–1.93 (2H, m, H-5), 1.78-1.88 (2H, m, H-3), 1.67 (3H, s, H -7); 1.47–1.57 (3H, m, H-4), 1.22 (3H, s, H-10), 1.20 (3H, s, H-9). ^13^C-NMR (CDCl_3_, 125 MHz) δ (ppm): 118.45 (C-2), 134.02 (C-1), 45.01 (C-4), 23.98 (C-5), 31.02 (C-6), 23.34 (C-7), 27.46 (C-10), 72.74 (C-8), 26.89 (C-3), 26.29 (C-9). EI-MS *m/z* (%):136 (48), 121 (58), 107 (11), 95 (22), 93 (67), 81 (36), 79 (18), 68(27), 59 (100), 55 (16), 43 (32), 41 (20). The data matched with previous reports [40,51].

α-*Terpinyl acetate* (**4**, Figure 1). Colorless oil, ^1^H-NMR (CDCl_3_, 500 MHz) δ (ppm): 5.40 (1H, brs, H-2), 2.06 (1H, s, H-4), 1.99-2.03 (2H, m, H-6), 2.00 (1H, m, H-3b), 1.98 (3H, s, H-12), 1.86 (1H, m, H-3a), 1.81 (1H, m, H-5b), 1.67 (3H, s, H-7), 1.46 (3H, s, H-10), 1.43 (3H, s, H-9), 1.31 (1H, m, H-5a). ^13^C-NMR (CDCl_3_, 125 MHz) δ (ppm): 170.78 (C-11), 133.98 (C-1), 120.32 (C-2), 84.86 (C-8), 42.57 (C-4), 30.89 (C-6), 26.38 (C-3), 23.86 (C-5), 23.31 (C-10), 3.35 (C-9), 23.13 (C-12), 22.50 (C-7). EI-MS *m/z* (%): 196 [M^+^, 5], 136 (58), 121 (100), 105 (22), 93 (86), 91 (46), 77 (26), 67 (37), 51 (21). The data matched with previous reports [51,52].

### 3.7. Contact Toxicity with Treated Filter Paper

Contact toxicity of the essential oil of *A. rupestris* aerial parts against *L. bostrychophila* was measured as described by Liu *et al.* [1]. Range-finding studies were run to determine the appropriate testing concentrations of the essential oil of *A. rupestris* and pure compounds. The essential oil and compound were diluted in acetone. The filter paper with 3.5 cm in diameter (Whatman) was treated with 150 μL of the solution. Then the filter paper after treated with solid glue (Glue Stick, Jong Ie Nara Co., Ltd. Hong Kong) was placed in a Petri dish (3.5 cm in diameter) and 10 booklice were put on the filter paper by using a hair brush. The plastic cover with holes was put and all the Petri dishes were kept in incubators at 27–29 °C, 70%–80% r.h. for 24 h. Acetone was used as negative control and pyrethrum extract was used as a positive control. Six concentrations (1.0%, 1.2%, 1.5%, 1.8%, 2.2%, and 4.5%) and five replicates of each concentration were used in all treatments and controls. Mortality of insects was observed and the observed data were corrected for control mortality using Abbott’s formula. The results from all replicates were subjected to probit analysis using the PriProbit Program V1.6.3 to determine LD_50_ values [53]. Pyrethrum extract (25% pyrethrine I and pyrethrine II) was purchased from Fluka Chemie (Buchs, Switzerland).

### 3.8. Fumigant Toxicity

Fumigant toxicity of the essential oil of *A. rupestris* aerial parts against *L. bostrychophila* was determined as described by Zhao [54]. Range-finding studies were run to determine the appropriate testing concentrations of the pure compounds and *C. wenyujin* essential oil. A filter paper strip (3.5 cm × 1.5 cm) treated with 10 μL of an appropriate concentration of test essential oil/compound in acetone. The impregnated filter paper was then placed in the bottom cover of glass bottle of 250 mL. The insects, 10 adults in a small glass bottle (8 mL), were exposed for 24 h. Each concentration had five replicates. Six concentrations (0.80%, 1.0%, 1.8%, 2.7%, 4.0%, 6.0%) were used in all treatments and controls. All the treatments were replicated five times. Acetone was used as negative control and dichlorvos was used as a positive control. The observed mortality data were corrected for control mortality using Abbott’s formula. The LC_50_ values were calculated by using Probit analysis [53]. Positive control, dichlorvos (99.9%) was purchased from Aladdin Reagent Company (Shanghai, China).

### 3.9. Repellency

The repellency of *A. rupestris* essential oil against *L. bostrychophila* was determined as described by Zhang [48]. Petri dishes (6 cm in diameter) were used to confine *L. bostrychophila* during the experiment. The crude essential oil and the isolated compounds were diluted in acetone to three concentrations (13.0, 6.4, 3.2 nL/cm^2^). Filter paper (6 cm in diameter) was cut in half and 150 μL of each concentration was applied separately to half of the filter paper as uniformly as possible with a micropipette. The other half (control) was treated with 150 μL of absolute acetone. Both the treated half and the control half were then air dried to evaporate the solvent completely (10 s). A full disc was carefully remade by attaching the tested half to the negative control half with tape. Care was taken so that the attachment did not prevent free movement of insects from the one half to another, but the distance between the filter-paper halves remained sufficient to prevent seepage of test samples from one half to another. Each remade filter paper after treated with solid glue (Glue Stick, Jong Ie Nara Co., Ltd., location) was placed in a Petri dish with the seam oriented in one of four randomly selected different directions to avoid any insecticidal stimuli affecting the distribution of insects. Twenty insects were released in the center of each filter-paper disc and a cover was placed over the petri dish. Five replicates were used and the experiment was repeated for three times. Counts of the insects present on each strip were made after 2 h and 4 h. The percent repellency of each volatile oil/compound was then calculated using the formula:

PR (%) = [(Nc ‒ Nt)/(Nc + Nt)] × 100

where Nc was the number of insects present in the negative control half, Nt was the number of insects present in the treated half. Analysis of variance (ANOVA) and Tukey’s test were conducted by using SPSS 10 for Windows 98. Percentage was subjected to arcsine square-root transformation before ANOVA and Tukey’s tests. The averages were then assigned to different classes (0 to V) (Table 4) [55]. A commercial repellent, DEET (*N*, *N*-diethyl-3-methylbenzamide), was purchased from the National Center of Pesticide Standards (Shenyang, China) and used as a positive control.

**Table 4:** The scale to be assign repellency of the essential oil of *Artemisia rupestris* and its constituents.

Class	Percent repellency	Class	Percent repellency	Class	Percent repellency
0	>0.01 to <0.1	II	20.1–40	IV	60.1–80
I	0.1–20	III	40.1–60	V	80.1–100

## 4. Conclusions

The study indicates that the essential oil of *A. rupestris* aerial parts and its four constituent compounds have potential for development into natural insecticides/fumigants and repellents for control of insects in stored grains.
